# Development of duplex real-time PCR for the detection of WSSV and PstDV1 in cultivated shrimp

**DOI:** 10.1186/1746-6148-10-150

**Published:** 2014-07-05

**Authors:** Carlos A G Leal, Alex F Carvalho, Rômulo C Leite, Henrique C P Figueiredo

**Affiliations:** 1 Department of Veterinary Medicine, Federal University of Lavras, Lavras, Brazil; 2 AQUAVET, Laboratory of Aquatic Animal Diseases, School of Veterinary Medicine, Federal University of Minas Gerais, Belo Horizonte, Brazil; 3 AQUACEN, National Reference Laboratory for Aquatic Animal Diseases, Ministry of Fisheries and Aquaculture, Federal University of Minas Gerais, Belo Horizonte, Brazil

**Keywords:** Coinfection, Clinical sensitivity, Duplex qPCR, IHHNV, *Litopenaeus vannamei*, WSSV

## Abstract

**Background:**

The *White spot syndrome virus* (WSSV) and *Penaeus stylirostris penstyldensovirus 1* (previously named *Infectious hypodermal and hematopoietic necrosis virus*-IHHNV) are two of the most important viral pathogens of penaeid shrimp. Different methods have been applied for diagnosis of these viruses, including Real-time PCR (qPCR) assays. A duplex qPCR method allows the simultaneous detection of two viruses in the same sample, which is more cost-effective than assaying for each virus separately. Currently, an assay for the simultaneous detection of the WSSV and the PstDV1 in shrimp is unavailable. The aim of this study was to develop and standardize a duplex qPCR assay for the simultaneous detection of the WSSV and the PstDV1 in clinical samples of diseased *L. vannamei*. In addition, to evaluate the performance of two qPCR master mixes with regard to the clinical sensitivity of the qPCR assay, as well as, different methods for qPCR results evaluation.

**Results:**

The duplex qPCR assay for detecting WSSV and PstDV1 in clinical samples was successfully standardized. No difference in the amplification of the standard curves was observed between the duplex and singleplex assays. Specificities and sensitivities similar to those of the singleplex assays were obtained using the optimized duplex qPCR. The analytical sensitivities of duplex qPCR were two copies of WSSV control plasmid and 20 copies of PstDV1 control plasmid. The standardized duplex qPCR confirmed the presence of viral DNA in 28 from 43 samples tested. There was no difference for WSSV detection using the two kits and the distinct methods for qPCR results evaluation. High clinical sensitivity for PstDV1 was obtained with TaqMan Universal Master Mix associated with relative threshold evaluation. Three cases of simultaneous infection by the WSSV and the PstDV1 were identified with duplex qPCR.

**Conclusion:**

The standardized duplex qPCR was shown to be a robust, highly sensitive, and feasible diagnostic tool for the simultaneous detection of the WSSV and the PstDV1 in whiteleg shrimp. The use of the TaqMan Universal Master Mix and the relative threshold method of data analysis in our duplex qPCR method provided optimal levels of sensitivity and specificity.

## Background

The *White spot syndrome virus* (WSSV) and the *Penaeus stylirostris penstyldensovirus 1* (PstDV1) (previously named *Infectious hypodermal and hematopoietic necrosis virus*, IHHNV) are major viral pathogens of penaeid shrimp, and pose a significant threat to shrimp aquaculture worldwide [1]. Highly infectious, the WSSV has a large broad of hosts (more than 93 arthropods species described), and a cosmopolitan distribution, being reported in the majority of shrimp farming countries [2,3]. The WSSV outbreaks in the whiteleg shrimp *Litopenaeus vannamei* were characterized by high mortality rates, reaching until 100% mortality in 3 to 10 days [4,5]. By contrast, the PstDV1 causes a chronic disease in *L. vannamei* called runt deformity syndrome (RDS). The RDS causes cuticular deformities and retards growth, leading to lower production efficiency and reducing the market value of harvests by 10% to 50% [4,5].

Several methods, including histological examination, electron microscopy, in situ hybridization, and polymerase chain reaction (PCR) methods, have been developed for diagnosis of the WSSV [6-8], and the PstDV1 infections [9,10]. Real-time PCR (qPCR) assays have been developed to detect the WSSV [6,7] and the PstDV1 [11,12]. These qPCR methods have demonstrated higher sensitivities than those of the other diagnostic methods used for the detection of these shrimp viruses [13].

Currently, 53 scientific manuscripts and 11 patents describe different methods to diagnose WSSV according to Web of Science™ Database (Thompson Reuters, USA). However, there is no available qPCR assay for simultaneous detection of the WSSV and the PstDV1 in shrimp. A duplex qPCR method allows the simultaneous detection of two viruses in the same sample, which is more cost-effective than assaying for each virus separately [14]. It is particularly interesting in epidemiological surveys, surveillance, and eradication programs, which require the analysis of large number of samples. Methods less time consuming and inexpensive are essential for the economic and technical feasibility of these programs, mainly when they are supported by governments. In addition, a duplex qPCR assay could improve the diagnosis of co-infection cases caused by WSSV and PstDV1. It is a growing issue to the biosecurity of shrimp farming.

The aim of this study was to develop and standardize a duplex qPCR assay for the simultaneous detection of the WSSV and the PstDV1 in clinical samples of diseased *L. vannamei*. In addition, to evaluate the performance of the TaqMan Universal PCR Master Mix (TUMM; Applied Biosystems, Carlsbad, CA, USA) and the QuantiTect Virus (QVK; Qiagen, Chatsworth, CA, USA) master mix with regard to the clinical sensitivity of the qPCR assay, as well as, different methods for qPCR results evaluation.

## Methods

### Samples and DNA extraction

*L. vannamei* were collected during outbreaks of WSSV and PstDV1 infections at various shrimp farms in the Brazilian states of Santa Catarina, Bahia, Rio Grande do Norte, and Pernambuco between 2004 and 2013. The samples were preserved in 96% ethanol, and immediately stored in the laboratory. A total of 43 samples were evaluated, among which 28 were WSSV-positive, five were PstDV1-positive, and ten were virus-negative. The WSSV- and PstDV1-positive shrimp showed clinical signs of white spot disease (WSD) and RDS, respectively, and the diagnosis was confirmed using conventional PCR methods [15]. According to the Ethics Committee in Animal Experimentation of Federal University of Minas Gerais (CEUA/UFMG, Brazil), this work did not need ethics approval, since evaluated dead animals sampled during outbreaks.

Total DNA was extracted from three of the left abdominal pleopods of each shrimp using the Wizard® DNA Genomic Purification (Promega, Madison, WI, USA). The purified DNA was quantified spectrophotometrically using a NanoVue spectrophotometer (GE Healthcare, Waukesha, WI, USA), and stored at −20°C.

### Plasmids, primers and probes

Purified plasmids containing the genomic sequences U50923 of WSSV and AF218266 of PstDV1 were acquired from OIE Reference Laboratory (University of Arizona’s Aquaculture Pathology Laboratory, USA), and used as positive controls. Ten-fold serial dilutions were used to construct the standard curve for the qPCR analysis. Plasmid amounts ranging from 2 to 2 × 10^5^ copies were used to determine the sensitivity of the assays. The primers and probes used for the qPCR have been described previously [6,16]. All of the primers used were purchased from Integrated DNA Technologies (Coralville, IA, USA). The WSSV hybridizing probe was labeled with 6-carboxy-fluorescein (FAM) at the 5' terminus and the dark quencher BHQ1 at the 3' terminus The PstDV1 hybridizing probe was labeled with dichloro-dimethoxyfluorescein (JOE) at the 5' terminus and BHQ1 at the 3' terminus. The probes were purchased from Sigma-Aldrich (St. Louis, MO, USA).

### Duplex qPCR Standardization

For the duplex qPCR standardization, a set of standard curves was constructed using six dilutions of each control plasmid to determine the optimal primer-probe concentrations for each reaction. For the duplex qPCR, the primers were evaluated at concentrations ranging from 5 to 100 pmol per reaction, and the probes were varied from 5 to 75 pmol per reaction. The standard curves were evaluated in triplicate for each qPCR mixture tested. The optimal reaction conditions were determined based on the following criteria: a slope factor of −3.099 to −3.59, corresponding to a PCR efficiency of 90% to 110%; a correlation coefficient > 0.99; and a lower average quantification cycle (*C*_q_) for each dilution [17]. A 6 × 6 checkerboard validation scheme was used to evaluate the potential for the cross-interaction of the WSSV and PstDV1 DNA templates in the duplex qPCR. The reaction results and the inhibitory effect were effectively monitored when the concentrations of the template DNA (control plasmid) ranged between 2 and 2 × 10^4^ copies per reaction.

### Clinical Sensitivity

The clinical sensitivity of the duplex qPCR assay was compared to that of separate singleplex assays for each virus using DNA samples extracted from WSSV-positive and PstDV1-positive shrimp, and the specificity analysis included ten virus-negative samples. The performance of the QVK and TUMM master mixes were also evaluated, according to the manufacturer's recommendations. The singleplex and duplex qPCR assays were conducted using standardized concentrations of each primer set and the specific probes and 50 ng of sample DNA in a 25-μL reaction volume.

The analysis of the PCR products was performed using a ViiA 7 Real-time PCR System (Life Technologies). The qPCR cycling protocol was the same for both kits, except that the initial denaturation step was 95°C for 5 min for the QVK and 95°C for 10 min for the TUMM. Data acquisition and analysis were performed using the ViiA 7 Software (Life Technologies, USA) using standard parameters with ROX normalization.

### qPCR Data analysis

The qPCR data were evaluated using two different methods. The first method was based on cycle threshold and *C*_q_ determinations, according to the guidelines of the *Minimum Information for Publication of Quantitative Real-Time PCR Experiments*[18,19], and the other method used the relative threshold algorithm of the ViiA 7 software to determine the cycle relative threshold (CRT) of each sample. A McNemar's chi-squared test was used to determine statistical differences between the sensitivities of the single and duplex qPCR methods, as well as those obtained using the different qPCR master mixes. All statistical analyses were performed using the R statistical software, as described previously [20]. The level of statistical significance was set at *P* < 0.05.

## Results

### qPCR Standardization

**Figure 1:**
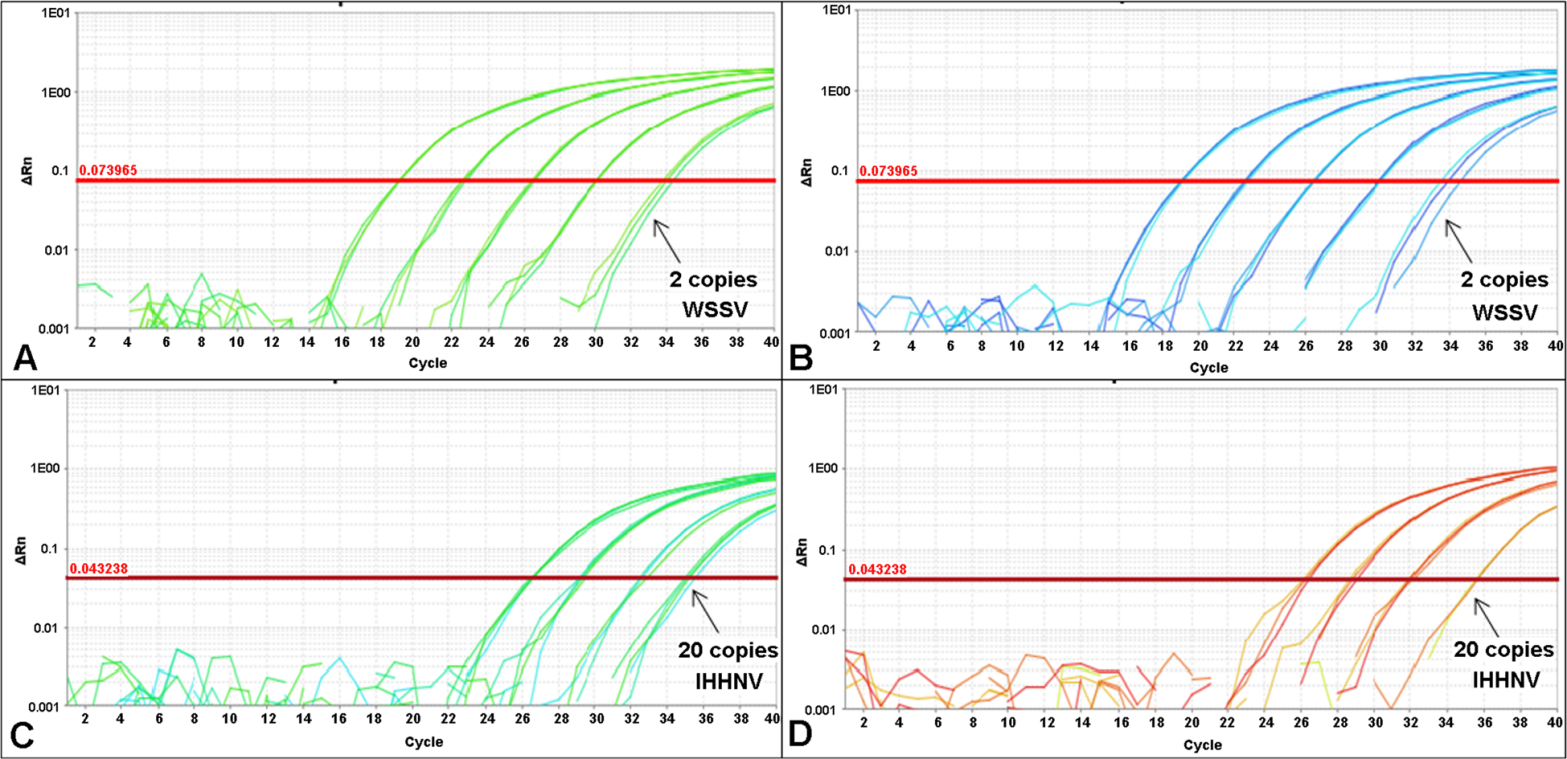
**Amplification plots of standard curves of singleplex and duplex qPCR.** Standard curves of serial dilution from 2 × 10^4^ to 2 copies of control plasmids showing the detection limit of two copies for WSSV in singleplex **(A)** and duplex format **(B)**, and 20 copies for PstDV1 in singleplex **(C)** and duplex format **(D)**.

We standardized the duplex qPCR assay for detecting WSSV and PstDV1 in clinical samples of diseased whiteleg shrimp. The optimal primer-probe concentrations were 60 pmol of each primer and 25 pmol of probe per reaction for both the singleplex and duplex qPCR assays. No difference in the amplification of the standard curve was observed between the duplex and singleplex assays (Figure 1), regardless of the master mix used. The mean (*n* = 3) PCR efficiencies of the singleplex (WSSV = 91%, PstDV1 = 90%) and duplex (WSSV = 91%; PstDV1 = 101%) assays that used the TUMM were within the optimal recommended range. The mean (*n* = 3) PCR efficiencies for the singleplex (WSSV = 96%, PstDV1 = 98%) and duplex (WSSV = 109%, PstDV1 = 109%) assays that used the QVK master mix were also within the optimal recommended range. The detection limits were two copies of WSSV control plasmid and 20 copies of PstDV1 control plasmid per reaction for both the singleplex and duplex assays (Figure 1).

The potential cross-interaction of the different DNA templates and its interference with PCR efficiency were evaluated using a 6 × 6 checkerboard analysis. The amplification plots of the WSSV and PstDV1 control plasmids were not significantly affected in mixtures with concentrations between 2 × 10^1^ and 2 × 10^4^ copies per reaction. At the highest PstDV1 plasmid concentration (2 × 10^5^ copies per reaction) the detection limit of the WSSV plasmid increased from two to 20 copies. By contrast, the detection limit of the PstDV1 plasmid was not affected by any of the WSSV concentrations tested.

### Clinical sensitivity

In the cycle threshold evaluation, no significant difference (*P* = 0.9265) in clinical sensitivity or specificity was observed between the singleplex assays for WSSV and PstDV1 and the duplex assays that used the same master mix (Table 1). The TUMM assays demonstrated superior clinical sensitivity for WSSV detection. However, no significant difference in sensitivity for PstDV1 detection was observed between the TUMM and QVK master mixes, with both products demonstrating a feasible clinical sensitivity for PstDV1 detection. The standardized duplex qPCR confirmed the presence of viral DNA in 28 samples that had previously tested positive for WSSV or PstDV1. The *C*_q_ values in the TUMM assay were lower than those for the QVK assay. An average reduction in *C*_q_ values of 3.11 was confirmed in the WSSV singleplex assay, and a 4.98 average reduction in *C*_q_ values was observed in the IHHNV singleplex assay. The TUMM duplex qPCR assays for WSSV and PstDV1 had 2.89 and 4.83 lower average *C*_q_ values, respectively. One case of coinfection was identified.

**Table 1:** Clinical sensitivities of qPCR assays for the detection of the WSSV and the PstDV1

Sample*	**Singleplex sensitivity (%)**		**Duplex sensitivity (%)**		**Coinfections (Duplex qPCR)**	
	**TaqMan**	**QuantiTect**	**TaqMan**	**QuantiTect**	**TaqMan**	**QuantiTect**
**Cycle threshold (*C***_**q**_**)**						
WSSV positive (n =28)	26 (92.85)	24 (85.71)	24 (85.71)	23 (82.14)	1	1
PstDV1 positive (n = 5)	2 (40)	2 (40)	2 (40)	2 (40)		
Negative (n = 10)	0	0	0	0		
**Relative threshold (CRT)**						
WSSV positive (n =28)	24 (85.71)	24 (85.71)	28 (100)	28 (100)	3	3
PstDV1 positive (n = 5)	2 (40)	5 (100)	5 (100)	5 (100)		
Negative (n = 10)	0	0	0	13**		

*Only one of the viruses had been previously detected in each sample.

**3 were WSSV-positive and 10 were PstDV1-positive.

The use of the relative threshold algorithm for data analysis significantly increased the clinical sensitivity of PstDV1 detection (*P* = 0.0026; Table 1). However, no significant difference in sensitivity for WSSV detection was observed (*P* = 1.0). Using the relative threshold algorithm for data analysis, the singleplex and duplex QVK assays demonstrated a higher level of clinical sensitivity (100% for both viruses) than the TUMM assays, compared with those determined using the cycle threshold algorithm. However, 13 false-positive results, 3 false-positive for WSSV and 10 false-positive for PstDV1, were obtained in the QVK duplex qPCR assay. No significant difference in clinical sensitivity was observed between the singleplex and duplex QVK qPCR assays. However, the use of the relative threshold algorithm for the analysis of the data for the QVK duplex qPCR reduced the specificity of the assay from 100% to 43.47%, compared with the specificities obtained using the cycle threshold algorithm. Three coinfections were identified in both the QVK and TUMM duplex assays using the relative threshold algorithm.

## Discussion

A duplex qPCR was standardized for WSSV and PstDV1 detection in clinical samples using primers and probes that have been previously validated [6,16]. Specificities and sensitivities similar to those of the singleplex assays were obtained using the optimized duplex qPCR conditions, allowing the identification of both singular and coinfections in diseased whiteleg shrimp. The simultaneous identification of the WSSV and the PstDV1 is useful because such coinfections in shrimp are common [9,21].

The analytical sensitivity of the duplex qPCR assay was similar to those singleplex for WSSV and PstDV1 assays and those obtained in previous studies [6,16]. The IHHNV detection limit of standardized duplex qPCR assay was similar to that reported for a real-time multiplex PCR method in a previous study [9]. However, this duplex qPCR assay detected 10- and 627-fold lower amounts of PstDV1 DNA than the previously described conventional duplex and multiplex PCR methods [21,22]. In addition, the WSSV detection limit for duplex qPCR assay was 1-fold lower than that obtained using previously described multiplex PCR methods [21,22], and 10 copies lower than that obtained using a previously described multiplex qPCR method [9]. These data demonstrate the superior performance of standardized duplex qPCR method, compared with other conventional and real-time PCR-based methods. In addition, the lower detection limit of standardized duplex qPCR assay makes it a clinically useful tool for identifying animals with a low viral load, such as that observed in shrimp larvae and post-larvae [23], reducing the potential for false negative results.

Data from qPCR analyses have commonly been evaluated using a fit point method, which uses a fixed threshold based on the difference in baseline fluorescence intensities to determine the *C*_q_ value of each sample. The qPCR efficiency is affected by multiple factors that can lead to significant quantification problems [24]. Such confounders are particularly relevant to qPCR-based clinical diagnostics for aquatic animal diseases because some inhibitors can contaminate the DNA samples from the tissues of certain species [25]. Sigmoidal-fit models can reduce these types of inaccuracies because their algorithms analyze each strain separately.

In the present analysis of the various qPCR methods, the use of the relative threshold algorithm, which is based on a sigmoidal-fit model, substantially improved the performance of both the singleplex and duplex qPCR assays, and the clinical sensitivity was increased from 40% to 100%. Inhibitors of qPCR are a common issue in diagnostics for crustaceans [25,26], leading to frequent misdiagnoses. The superior performance of TUMM duplex qPCR assay should resolve such problems with regard to the detection of the WSSV and the PstDV1, while reducing false negative results. The QVK master mix contains an optimized combination of potassium chloride, ammonium sulfate, and a synthetic factor that enhance primer annealment. However, this may also cause nonspecific annealing in some cases, the type of which the present results and those of previous studies have confirmed [26,27].

Using the duplex qPCR assay, three cases of WSSV- PstDV1 coinfection were identified, for which the animals presented with clinical signs of one disease only. Using a duplex PCR method, Yang et al. [21] were the first to describe a WSSV- PstDV1 coinfection in *L. vannamei*. Xie et al. [9] also identified one case of WSSV- PstDV1 coinfection among 15 animals that were screened using a multiplex qPCR method. The standardized duplex qPCR assay identified a higher frequency of WSSV- PstDV1 coinfections than those reported in previous studies, which might be attributed to its superior sensitivity, compared with that of these previously described methods. This may also indicate that the frequency of natural coinfection in Brazilian shrimp farms is higher than that of other countries. Previous studies demonstrated that WSSV and PstDV1 infections occurred in shrimp cultivated in Brazil and other countries of South America [3,10,25,28,29]. However, future studies have to be performed to address the frequency of WSSV/PstDV1 coinfection in shrimp farms of the region.

## Conclusions

The standardized duplex qPCR is a robust and highly sensitive diagnostic tool for the simultaneous detection of the WSSV and the PstDV1 in whiteleg shrimp. The use of the TUMM and the relative threshold method of data analysis in WSSV/ PstDV1 duplex qPCR method provided optimal levels of sensitivity and specificity.

## Competing interests

The authors declare that they have no competing interests.

## Authors’ contributions

CAGL conceived and designed the study, carried out the methods, analyzed the data, and drafted the article. AFC collaborated in the execution of molecular methods. RCL participated in coordination and management of the study. HCPF participated in study design, coordination, and edition of article. All authors read and approved the final manuscript.
